# The association of *E2F1* and *E2F2* single nucleotide polymorphisms with laryngeal squamous cell carcinoma pathomorphological features

**DOI:** 10.1186/s12885-024-11953-z

**Published:** 2024-02-15

**Authors:** Tomas Jakstas, Agne Bartnykaite, Evaldas Padervinskis, Aurelija Vegiene, Elona Juozaityte, Virgilijus Uloza, Rasa Ugenskiene

**Affiliations:** 1 Department of Otorhinolaryngology, Lithuanian University of Health Sciences, Kaunas, Lithuania; 2 Oncology Research Laboratory, Oncology Institute, Lithuanian University of Health Sciences, Kaunas, Lithuania; 3 Oncology Institute, Lithuanian University of Health Sciences, Kaunas, Lithuania; 4 Department of Genetics and Molecular Medicine, Lithuanian University of Health Sciences, Kaunas, Lithuania

**Keywords:** E2F1, E2F2, SNP, Laryngeal squamous cell carcinoma

## Abstract

**Background:**

Laryngeal squamous cell carcinoma (LSCC) is one of the most common types of cancer in the upper respiratory tract. It is well-known that it has a high mortality rate and poor prognosis in advanced stages. There are well-known risk factors for LSCC, though new specific and prognostic blood-based markers for LSCC development and prognosis are essential. The current study aimed to evaluate the impact of four different single nucleotide polymorphisms (SNPs), *E2F1* (rs3213183 and rs3213180) and *E2F2* (rs2075993 and rs3820028), on LSCC development, morphological features, and patient 5-year survival rate.

**Methods:**

A total of 200 LSCC patients and 200 controls were included in this study; both groups were matched by age and sex. In the present study, we analyzed four single nucleotide polymorphisms (SNPs) in the genes *E2F1* (rs3213183 and rs3213180) and *E2F2* (rs2075993 and rs3820028) and evaluated their associations with the risk of LSCC development, its clinical and morphological manifestation, and patients 5-year survival rate. Genotyping was carried out using RT-PCR.

**Results:**

None of the analyzed SNPs showed a direct association with LSCC development. *E2F2* rs2075993 G allele carriers (OR = 4.589, 95% CI 1.050-20.051, *p* = 0.043) and rs3820028 A allele carriers (OR = 4.750, 95% CI 1.088–20.736, *p* = 0.038) had a statistically significantly higher risk for poor differentiated or undifferentiated LSCC than non-carriers. *E2F1* rs3213180 GC heterozygotes were found to have a 3.7-fold increased risk for lymph node involvement (OR = 3.710, 95% CI 1.452–9.479, *p* = 0.006). There was no statistically significant association between investigated SNPs and patient 5-year survival rate.

**Conclusions:**

The present study indicates that *E2F2* rs2075993 and rs3820028 impact LSCC differentiation, whereas *E2F1* rs3213180 - on lymph node involvement.

**Supplementary Information:**

The online version contains supplementary material available at 10.1186/s12885-024-11953-z.

## Background

Laryngeal squamous cell carcinoma (LSCC) is a type of cancer that affects the larynx (voice box) and is one of the most common types of cancer that affects the upper part of the respiratory system. LSCC often has a high mortality rate and a poor prognosis for patients, making it a severe and potentially life-threatening condition. In 2020, 184,615 new cases of LSCC were diagnosed, and 99,840 patients died from this cancer worldwide [1]. According to the World Health Organization (WHO), the incidence of laryngeal cancer will rise from 184,615 in 2020 to 284,000 in 2040, with a mortality rate increasing from 52.4 to 64.5% by 2040 [2]. It is well-known that laryngeal cancer is more prevalent in males than in females, 3.6/100 000 and 0.49/100 000, respectively [2]. This is primarily associated with ill habits, such as smoking and high alcohol consumption, that males tend to have more often than females [3–5]. Although tobacco and alcohol consumption are of great significance in LSCC carcinogenesis, only a tiny part of smokers and drinkers are diagnosed with LSCC [6]. This suggests that environmental and genetic factors may contribute to LSCC development. This forces attention to blood-based genetic biomarkers to speed up diagnostics and develop new treatment options for LSCC patients [7]. Several studies have demonstrated the importance of specific gene variants, particularly in combination with substances such as alcohol and tobacco, that play a role in the development and prognosis of LSCC [7–10].

E2F1 and E2F2 are transcription factors that belong to the E2F family of proteins. These proteins play a critical role in regulating the cell cycle and are involved in controlling cell proliferation and cell death [11, 12], both processes being essential in carcinogenesis. E2F1 is found to play a role in a variety of cancer types, including breast [13], gastric [14], and colorectal cancer [15], and has been shown to promote cell proliferation and inhibit apoptosis. E2F2, on the other hand, has been shown to have a suppressive effect on cell proliferation and to promote apoptosis in specific cancer types [16, 17].

Single nucleotide polymorphisms (SNP) are potential markers for individual susceptibility to the disease. Analysis of SNP might be critical for the early diagnosis, prognosis, and individualized, targeted cancer treatment [18]. A limited number of studies in the literature have investigated the role of *E2F1* and *E2F2* SNPs in head and neck cancer. Earlier published studies suggest that specific SNPs may be associated with an increased risk of head and neck cancer [19–21]. However, there are no studies available on the effect of *E2F1* and *E2F2* SNPs on LSCC phenotype. Therefore, the purpose of this study was to investigate four SNPs in *E2F1* (rs3213183 and rs3213180) and *E2F2* (rs2075993 and rs3820028) genes and to determine their associations with the risk of LSCC development and their impact on cancer clinical and morphological manifestation and patients 5-year survival rate.

## Methods

### LSCC group

A thorough otorhinolaryngological examination with flexible endoscopy and/or video laryngostroboscopy was performed for all LSCC patients at the outpatient clinics of the Department of Otorhinolaryngology at Lithuanian University of Health Sciences (LUHS), Kaunas, Lithuania. Direct microlaryngoscopy with biopsy was performed for all patients. The diagnosis of LSCC was confirmed by histopathological testing at the Department of Pathology, LUHS. The final diagnosis of LSCC was based on clinical data and the results of histological examination and laryngeal and neck computed tomography (CT) with contrast enhancement or magnetic resonance imaging (MRI). The staging of LSCC was done following the Guidelines for Head and Neck Cancers Classification, Version 2.2020, accepted by the National Comprehensive Cancer Network (NCCN) [22]. Patients diagnosed with/or suspected of having other types of cancer and individuals under 18 years of age were excluded from this study. The exclusion of female participants from this study was based on the fact that LSCC is more prevalent in men [2], and the incidence of LSCC in women is extremely low, with only a few cases reported nationwide annually. Clinical data were obtained through personal interviews during counseling and by reviews of patients’ case records. For SNP analyses peripheral venous blood samples were collected.

In total, a group of 200 men who were diagnosed with LSCC were consequently enrolled in this retrospective case-control study. Information about tumor size (T), nodal metastasis (N), distant metastasis to other organs (M), clinical stage (ST), and cancer cell differentiation grade (G) was collected. We divided T, N, M stages, ST and G into two groups: T1-2 (low stage) versus (VS) T3-4 (high stage), N0 (lymph nodes without metastasis) VS N1-3 (lymph nodes with metastasis), M0 (no distant metastasis) VS M1 (with distant metastasis), ST 1–2 (early stage) VS ST 3–4 (advanced stage) and G1-2 (well/moderately differentiated cancers) VS G3-4 (poor/undifferentiated cancers) [23, 24].

### Reference group

The reference group consisted of 200 men recruited during an annual health checkup in an outpatient clinic. Patients who were treated for cancer or were suspected to have any oncologic disease - were excluded from the study.

The LSCC patients and reference groups were matched by age (Table 1) and sex (males only). The study was approved by the Kaunas Regional Biomedical Research Ethics Committee (authorization protocols No. BE-2-37, No. BE-2-10, and No. P1-BE-2-10/2014), and informed consent was obtained from all the participants prior to inclusion in the study.

### Selection of SNPs

The investigated SNPs were selected according to the following criteria: >5% minimal allele frequency (MAF) in the 1000 Genomes or HapMap databases (https://www.ncbi.nlm.nih.gov/snp/) and previously defined associations with head and neck or other types of cancer susceptibility [25–27].

### Sample size

To determine the appropriate sample size for this study, the formula *n = Z*^*2*^*×p×(1 − p)/E*^*2*^ was used, where *n* represents the necessary sample size, *Z* is the 95% confidence level, p is the minimal allele frequency, and *E* is the margin of error. The minimal allele frequency was obtained from the SNP database and ranged from 11 to 34%. The margin of error was set at 8%. Based on these parameters, the sample size was calculated to be between 60 and 164 patients.

### Genotyping of SNPs

The genotyping of *E2F1* rs3213183 and rs3213180, and *E2F2* rs2075993 and rs3820028 was carried out at the Oncology Research Laboratory of Oncology Institute at LUHS. Venous blood samples for DNA extraction were collected in ethylenediamine tetra-acetic acid tubes. According to the manufacturer’s recommendations, genomic DNA from peripheral blood leucocytes was extracted using a DNA purification kit (Thermo Fisher Scientific, Waltham, MA, USA). SNP rs3213183 and rs3213180 in *E2F1*, and rs2075993 and rs3820028 in *E2F2* genes were estimated by using commercially available TaqMan (Thermo Fisher Scientific Baltics, Vilnius, Lithuania) genotyping kits using real–time PCR (RT-PCR). Statistical analysis was performed using IBM Statistical Package for the Social Sciences (SPSS) version 29.0 (SPSS Inc., Chicago, IL, USA).

### Quality control of genotyping

5% (5%) of randomly chosen samples were repetitively analyzed to confirm the results of initial genotyping.

### Survival rate

The LSCC group data about the 5-year survival rate and the cause of death were collected from the Lithuanian State Register of Death Cases and Their Causes.

### Statistical analysis

Differences in genotype frequencies among the groups were evaluated by the Hardy–Weinberg equilibrium (HWE) using a chi-square test (*p >* 0.05). The associations between SNP genotypic and allelic models and pathomorphological characteristics were analyzed using Pearson’s chi-square or Fisher’s exact tests. Univariate logistic regression was used to calculate the odds ratios (ORs) with a 95% confidence interval (CI) to estimate each SNP’s impact on pathomorphological characteristics and determine whether it increases or decreases the risk. The multivariate logistic regression model was used to estimate the adjusted ORs for statistically significant results in the univariate logistic regression. For multiple comparisons Bonferroni correction was applied. The difference was considered statistically significant when the *p*-value was less than 0.05 (Bonferroni-corrected *p* < 0.05). Relationships between studied SNPs and overall survival (OS) were assessed for survival analysis. Survival curves were generated using the Kaplan–Meier method and compared using log-rank, Breslow, and Tarone-Ware tests.

## Results

The study included 200 male patients with LSCC, with a median age of 64.5 years (49–85). Additionally, 200 healthy male subjects with a median age of 65.5 years (50–75) were included as controls. Statistical analysis revealed no significant difference in age between the case and control groups (*p* = 0.073). Table 1 provides detailed demographic characteristics of the entire study population. The enrollment process started in 2016, and the cases enrolled in the study are from 2016 until 2023. The range of follow-up time was from 1 to 60 months. The mean average of the follow-up time was 39 months, and the median of the follow-up time was 30 months.

**Table 1:** Demographic characteristics of the study population

Cases (*n* = 200) age median (min-max) in years.	64.50 (49–85) *
Controls (*n* = 200) age median (min-max) in years.	65.50 (50–75) *
T1-2, n (%)	102 (51.0)
T3-4, n (%)	98 (49.0)
N0, n (%)	134 (67.0)
N1-3, n (%)	66 (33.0)
M0, n (%)	197 (98.5)
M1, n (%)	3 (1.5)
G1-2, n (%)	166 (83.0)
G3-4, n (%)	34 (17.0)
ST I-II, n (%)	97 (48.5)
ST III-IV, n (%)	103 (51.5)
*P = 0.073	

Abbreviations: T1-2 - small tumor, T3-4 - large tumor, N0 - lymph nodes without metastasis, N1-3 - lymph nodes with metastasis, M0 - no distant metastasis to other organs, M1- distant metastasis present, ST I-II - early clinical stage, ST III-IV - advanced clinical stage, G1-2 - well/moderately differentiated cancers, G3-4 - poor/undifferentiated cancers

The distribution of the examined SNPs *E2F1* rs3213183, *E2F2* rs2075993, and rs3820028 was in accordance with the HWE (*p* > 0.05). Although *E2F1* rs3213180 did not adhere to the HWE (*p* = 0.019), we chose not to exclude this SNP from the subsequent analysis. It was determined that there were no significant differences between the distribution of analyzed SNP genotypes and alleles in LSCC and control groups (Table 2). The results suggest that the analyzed SNPs are not associated with the increased risk for LSCC development.

**Table 2:** SNP genotype and allele distribution in LSCC and control groups

Gene	SNP	Genotype	Frequency		***P*** value	OR (95%CI)	***P*** value
			Cases	Controls			
*E2F1*	rs3213183	GG	110 (55.0)	118 (59.0)	0.661	Reference	
		GA	78 (39.0)	69 (34.5)		1.213 (0.801–1.837)	0.363
		AA	12 (6.0)	13 (6.5)		0.990 (0.433–2.263)	0.981
		A allele non-carriers	110 (55.0)	118 (59.0)	0.480	Reference	
		A allele carriers	90 (45.0)	82 (41.0)		1.177 (0.792–1.750)	0.419
		G allele non-carriers	12 (6.0)	13 (6.5)	1.000	Reference	
		G allele carriers	188 (94.0)	187 (93.5)		1.089 (0.484–2.449)	0.836
	rs3213180	GG	174 (87.0)	168 (84.0)	0.096	Reference	
		GC	21 (10.5)	31 (15.5)		0.654 (0.361–1.184)	0.161
		CC	5 (2.5)	1 (0.5)		4.828 (0.558–41.755)	0.153
		C allele non-carriers	174 (87.0)	168 (84.0)	0.478	Reference	
		C allele carriers	26 (13.0)	32 (16.0)		0.784 (0.448–1.372)	0.395
		G allele non-carriers	5 (2.5)	1 (0.5)	0.215	Reference	
		G allele carriers	195 (97.5)	199 (99.5)		0.196 (0.023–1.693)	0.138
*E2F2*	rs2075993	AA	39 (19.5)	49 (24.5)	0.388	Reference	
		AG	102 (51.0)	101 (50.5)		1.269 (0.768–2.097)	0.353
		GG	59 (29.5)	50 (25.0)		1.483 (0.843–2.608)	0.172
		G allele non-carriers	39 (19.5)	49 (24.5)	0.277	Reference	
		G allele carriers	161 (80.5)	151 (75.5)		1.340 (0.833–2.155)	0.228
		A allele non-carriers	59 (29.5)	50 (25.0)	0.369	Reference	
		A allele carriers	141 (70.5)	150 (75.0)		0.797 (0.512–1.239)	0.313
	rs3820028	GG	40 (20.0)	52 (26.0)	0.297	Reference	
		GA	101 (50.0)	99 (49.5)		1.326 (0.807–2.180)	0.265
		AA	59 (29.5)	49 (24.5)		1.565 (0.894–2.740)	0.117
		A allele non-carriers	40 (20.0)	52 (26.0)	0.191	Reference	
		A allele carriers	160 (80.0)	148 (74.0)		1.405 (0.879–2.246)	0.155
		G allele non-carriers	59 (29.5)	49 (24.5)	0.311	Reference	
		A allele carriers	141 (70.5)	151 (75.5)		0.776 (0.498–1.208)	0.261

The subsequent analysis of the LSCC study group revealed statistically significant associations between *E2F2* rs2075993, *E2F2* rs3820028 polymorphisms, and LSCC differentiation grade (*p* = 0.031 and 0.032, respectively). Furthermore, a significant association was found between the *E2F1* rs3213180 polymorphism and tumor size (T) (*p* = 0.039), lymph node involvement (N) (*p* = 0.005), and LSCC clinical stage (ST) (*p* = 0.008) (Table 3). That was followed by univariate logistic regression analysis. Only statistically significant results are mentioned below. It was determined that individuals carrying the A allele of *E2F2* rs3820028 (OR = 4.750, 95% CI 1.088–20.736, *p* = 0.038) and individuals carrying the G allele of *E2F2* rs2075993 (OR = 4.589, 95% CI 1.050-20.051, *p* = 0.043) had an increased risk for poorly differentiated or undifferentiated LSCC compared to the non-carriers. Statistically significant results were observed in the univariate logistic regression model for *E2F1* rs3213180 GC heterozygotes, impacting lymph node involvement (OR = 3.710, 95% CI 1.452–9.479, *p* = 0.006) (Table 3).

**Table 3:** SNPs association with pathomorphological LSCC parameters (bold text highlighting statistically significant results)

Variable / SNP		Frequency, n (%)		***P*** value*	OR (95%CI)	***P*** value
*Histological grade (G)*		G1-2	G3-4			
rs2075993	AA	37 (22.3)	2 (5.9)	0.088	Reference	
	AG	82 (49.4)	20 (58.8)		4.512 (1.002–20.313)	0.050
	GG	47 (28.3)	12 (35.3)		4.723 (0.995–22.426)	0.051
	G allele non-carriers	37 (22.3)	2 (5.9)	**0.031**	Reference	
	G allele carriers	129 (77.7)	32 (94.1)		4.589 (1.050-20.051)	**0.043**
	A allele non-carriers	47 (28.3)	12 (35.3)	0.416	Reference	
	A allele carriers	119 (71.7)	22 (64.7)		0.724 (0.332–1.580)	0.417
rs3820028	GG	38 (22.9)	2 (5.9)	0.078	Reference	
	GA	81 (48.8)	20 (58.8)		4.691 (1.043–21.105)	**0.044**
	AA	47 (28.3)	12 (35.3)		4.851 (1.023–23.012)	**0.047**
	A allele non-carriers	38 (22.9)	2 (5.9)	**0.032**	Reference	
	A allele carriers	128 (77.1)	32 (94.1)		4.750 (1.088–20.736)	**0.038**
	G allele non-carriers	47 (28.3)	12 (35.3)	0.416	Reference	
	G allele carriers	119 (71.7)	22 (64.7)		0.724 (0.332–1.580)	0.417
*Tumor size (T)*		T1-2	T3-4			
rs3213180	GG	89 (87.3)	85 (86.7)	**0.039**	Reference	
	GC	8 (7.8)	13 (13.3)		1.701 (0.672–4.310)	0.262
	CC	5 (4.9)	0 (0.0)		x	
	C allele non-carriers	89 (87.3)	85 (86.7)	0.913	Reference	
	C allele carriers	13 (12.7)	13 (13.3)		1.047 (0.459–2.387)	0.913
*Lymph node involvement (N)*		N0	N1-3			
rs3213180	GG	121 (90.3)	53 (80.3)	**0.005**	Reference	
	GC	8 (6.0)	13 (19.7)		3.710 (1.452–9.479)	**0.006**
	CC	5 (3.7)	0 (0.0)		x	
	C allele non-carriers	121 (90.3)	53 (80.3)	**0.048**	Reference	
	C allele carriers	13 (9.7)	13 (19.7)		2.283 (0.992–5.256)	0.052
*Stage (ST)*		ST I-II	ST III-IV			
rs3213180	GG	86 (88.7)	88 (85.4)	**0.008**	Reference	
	GC	6 (6.2)	15 (14.6)		2.443 (0.906–6.590)	0.078
	CC	5 (5.2)	0 (0.0)		x	
	C allele non-carriers	86 (88.7)	88 (85.4)	**0.025**	Reference	
	C allele carriers	11 (11.3)	15 (14.6)		1.333 (0.579–3.065)	0.499

* - Chi-square test or Fisher’s exact test; x - not applicable

When the results were adjusted for other characteristics such as patient age, tumor size, and histological grade in multivariate logistic regression analysis, rs3213180 GC genotype remained statistically significantly associated with unfavorable outcomes related to nodal metastasis (OR = 4.268, 95% CI 1.310-13.905, *p* = 0.016) (Table 4).

**Table 4:** Impact of rs3213180 on nodal metastasis. Multivariate logistic regression model (bold text highlighting statistically significant results)

Feature	Variables	OR	95%CI	***P*** value
N1-3 versus N0	rs3213180 (GC vs. GG)	4.268	1.310-13.905	**0.016**
	T (T3-4 vs. T1-2)	16.379	6.906–38.848	**< 0.001**
	G (G3-4 vs. G1-2)	1.699	0.685–4.212	0.253
	Age	0.994	0.948–1.043	0.809

LSCC patient 5-year survival was measured by log-rank, Breslow, and Tarone-Ware tests. Survival curves were generated using the Kaplan–Meier method. There were no significant differences in the survival of patients with different genotypes (Supplementary Materials: Figure S1– S4, Table S1-4). Our data suggest that the analyzed SNPs do not contribute to the 5-year survival rate of LSCC patients.

## Discussion

The E2F1 and E2F2 genes are both members of the E2F gene group. They are transcriptional factors that are well-known to affect cell fate and govern cancer development [11] by regulating cell proliferative and antiproliferative processes [12]. Although the exact mechanism underlying *E2F*-related tumor susceptibility is not fully understood with conflicting data available [26, 28, 29], *E2F1* and *E2F2* SNPs have been shown to influence the risk of various cancers such as head and neck squamous cell carcinoma [19, 30, 31], breast [32], colon and colorectal [16, 33, 34], gastric [35], ovarian [36] and other types of cancers [30]. However, as far as we know, no studies have shown the effect of polymorphisms in *E2F1* and *E2F2* genes on LSCC development or their impact on LSCC pathomorphological parameters.

This study examined E2F1 rs3213183, rs3213180, and E2F2 rs2075993, rs3820028 SNPs in patients with LSCC. Three of the mentioned SNPs are known to be located in the 3’untranslated region (UTR) (rs3213180, rs2075993, rs3820028) and one (rs3213183) in 5’UTR and were shown to have an impact on the head and neck or other types of cancer development [19, 25–27, 30]. Various studies have demonstrated that SNPs in the 5’UTR region can affect gene transcription and messenger RNA (mRNA) translation efficiency, resulting in altered protein levels. It is also known that SNPs in the 3’UTR, targeted by micro RNAs (miRNA), can affect RNA translation in this way, altering gene expression [37]. These mechanisms are widely recognized as potential for individual cancer risk [26, 37, 38].

The results of our study showed no statistically significant differences in genotype distribution between LSCC and reference groups. To our knowledge, the association between E2F1 rs3213183, rs3213180, E2F2 rs2075993, rs3820028, and LSCC has never been investigated. However, the effect of SNPs in the genes, as mentioned earlier, was analyzed in the head and neck cancers group. Lu et al., in their study, found that the combined effect of different SNPs may play a role in head and neck cancer development. They demonstrated a higher impact of SNPs on cancer development in younger male patients without smoking/alcohol consumption and with family cancer history in first-degree relatives [19]. It is important to mention that *the* study by Lu et al. demonstrated that a single SNP may not play a significant role in mediating personal risk for head and neck cancer, which concurs with our results.

The multifactorial etiopathogenesis of LSCC is well-known [39]. It is important to stress that different SNPs may contribute to various aspects of carcinogenesis either by altering the risk of cancer development, modifying the tumor phenotype, or affecting the cause of the disease. Various genetic alterations have been shown to impact the development and progression of LSCC in other studies [40–44]. Although the investigated SNPs did not affect LSCC development or patient 5-year survival, several significant associations with tumor phenotype were determined. As mentioned earlier, we demonstrated the importance of the SNPs on LSCC nodal metastasis and differentiation grade– the features associated with a more aggressive LSCC course. These findings might be helpful in treatment tactic selection in LSCC patients based on the individual genetic profile. It is worth mentioning that underlying biological mechanisms of the already identified genetic associations in other studies are still not fully understood and conflicting [35–49], indicating the importance of further research. Cancer researchers are looking for an ideal biomarker that would help diagnose cancer, detect metastases, assess tumor spread, and detect residual disease– though no such marker has been found by any of the research [50]. Despite this, data from our research provides new insights into LSCC pathogenesis and may be used in the future.

A recent study by Chen et al. found that E2F2 rs3820028 and E2F2 rs2075993 were associated with the risk of head and neck cancer development in the Chinese population [30]. Although we reached different conclusions based on our data, this difference might be because our study group included only a pure cohort of male LSCC patients. In contrast, *Chen et al.* investigated the whole group of head and neck cancers. Furthermore, our sample size was smaller, whereas *Chen et al.* investigated 679 patient and control pairs. Additionally, the frequencies of SNPs vary in distant populations, which might also contribute to the differences mentioned above.

There is a lack of literature on associations between *E2F1* and *E2F2* SNPs and LSCC pathomorphological characteristics. Our findings showed that *E2F2* rs2075993 G allele carriers and rs3820028 A allele carriers had a 4.5-fold and 4.7-fold higher risk for poorly differentiated or undifferentiated LSCC, respectively. The results suggest that these SNPs located in 3’UTR have a significant impact on LSCC differentiation that has not yet been demonstrated in the other studies. Furthermore, it is essential to mention that rs2075993 G > A alleles and rs3820028 A > G alleles are partially linked as GA and AG haplotypes were determined in the majority of our LSCC patients (in 199 out of 200).

A study by Yaoxu et al. investigated the expression of E2F genes in head and neck cancer patients and found that the expression levels of E2F1, E2F2, E2F5, E2F6, E2F7, and E2F8 in the N2 and N3 stages were significantly higher than those in the N0 and N1 stages [47]. Our study revealed that *the E2F1* rs3213180 GC genotype was significantly related to the development of lymph node metastasis in both univariate and multivariate analyses. The heterozygotes of rs3213180 had a 4-fold higher risk for nodal metastasis (univariate analysis: OR = 3.710, 95% CI 1.452–9.479, *p* = 0.006; multivariate analysis: OR = 4.268, 95% CI 1.310-13.905, *p* = 0.016) and the findings are consistent with results of *Yaoxu et al.*. Furthermore, rs3213180 SNP was earlier proven to alter *E2F1* gene expression [26]. This suggests that *E2F1* rs3213180, located in 3’UTR, plays a pivotal role in lymph node metastasis, resulting in a more aggressive LSCC phenotype predisposing patients to a higher clinical stage (ST).

The main strength of this study was the collection of a pure LSCC cohort, which was matched by age and sex with controls. Furthermore, all the samples were collected in a single hospital unit using a unified protocol for patient treatment, sample handling, and investigation. Moreover, significant findings of E2F2 rs2075993, rs3820028 involvement in LSCC differentiation, and the impact of E2F1 rs3213180 on lymph node metastasis were determined. It is worth mentioning that most of the earlier published studies provided data under the unified head and neck squamous cell carcinoma term [51]. In this way, all malignant tumors from different localizations (oropharyngeal, nasopharyngeal, hypopharyngeal, laryngeal, and sinonasal regions) were investigated as one disease. These studies did not consider that these malignancies have different etiologies, biological and clinical behaviors, prognoses, and distinct genetic predispositions [52, 53]. Moreover, we believe that investigating different localizations of head and neck cancers as one disease may leave possible meaningful associations for specific cancer types undetected. Therefore, pure cohort studies of single-location cancers are essential in head and neck cancer research.

Certain limitations of the present study must be addressed. A larger sample size could be beneficial. Additionally, the involvement of environmental factors should be considered - smoking and alcohol consumption habits were not investigated. However, this is a targeted task for future research.

The broad range of patient follow-up times (1 to 60 months) creates potential bias in drawing conclusions from our data. The mean average (39 months) and median (30 months) provide some general tendencies. However, due to the presence of a small number of patients with significantly extended follow-up times and a relatively low number of observed deaths (66 patients, comprising 33%), there is a possibility of skewness [54]. Therefore, conducting additional studies with extended follow-up periods is crucial to eliminate any possible bias and obtain conclusive data about the outcomes and patient 5-year survival rate.

## Conclusions

This study presents valuable insight into LSCC carcinogenesis as *E2F2* rs2075993 and rs3820028 were proven to play a role in LSCC differentiation, and *E2F1* rs3213180 in lymph node metastasis. The results suggest that the analyzed SNPs (E2F1 rs3213183, rs3213180, E2F2 rs2075993, and rs3820028) are not associated with an increased risk of LSCC development. The genotypic distribution of the mentioned SNPs does not influence the 5-year survival rate of LSCC patients, though further studies are needed with extended follow-up periods to draw definitive conclusions.

### Electronic supplementary material

Below is the link to the electronic supplementary material.

Supplementary Material 1

## Data Availability

The datasets generated during the current study are available from the corresponding author upon reasonable request.

## Abbreviations

LSCC: Laryngeal squamous cell carcinoma
WHO: World Health Organization
SNP: single nucleotide polymorphism
RT: PCR-real-time PCR
LUHS: Lithuanian University of Health Sciences
CT: Computed tomography
MRI: Magnetic resonance imaging
NCCN: National Comprehensive Cancer Network
T: tumor size
N: nodal metastasis
M: distant metastasis to other organs
ST: clinical stage
G: cancer cell differentiation grade
VS: versus
MAF: minimal allele frequency
SPSS: Statistical Package for the Social Sciences
HWE: Hardy-Weinberg equilibrium
OR: odds ratio
CI: confidence interval
OS: overall survival
UTR: untranslated region
mRNA: messenger RNA
miRNA: micro-RNA
