# Supplementary material for: The association of E2F1 and E2F2 single nucleotide polymorphisms with laryngeal squamous cell carcinoma pathomorphological features
Source: BMC Cancer. 2024 Feb 15;24:214. doi: 10.1186/s12885-024-11953-z (PMC10870611; doi:10.1186/s12885-024-11953-z)
Supplement: Supplementary file 1 — Supplementary Material 1 [file 12885_2024_11953_MOESM1_ESM.docx]

Supplementary Materials:


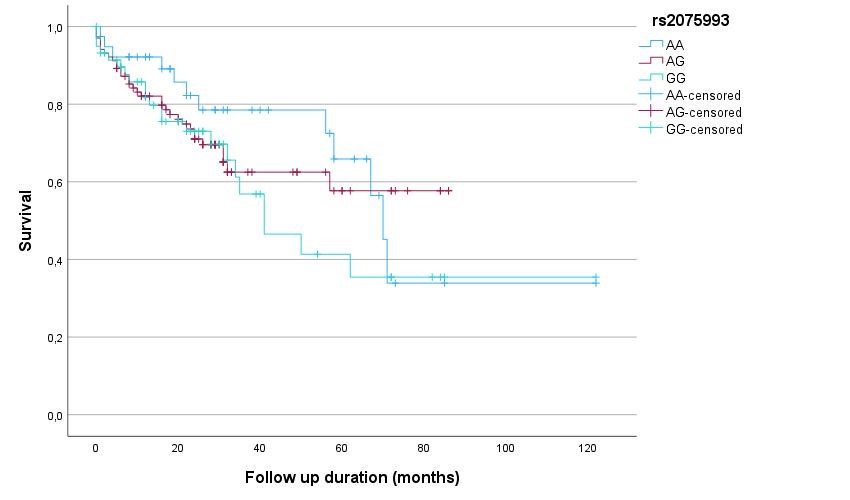


Figure S1. Five-year survival rate according to the distribution of *E2F2* rs2075993 genotypes.

|  | Chi-Square | df | p-value |
| --- | --- | --- | --- |
| Log Rank (Mantel-Cox) | 1.310 | 2 | 0.519 |
| Breslow (Generalized Wilcoxon) | 1.835 | 2 | 0.400 |
| Tarone-Ware | 1.720 | 2 | 0.423 |

Table S1. Log-rank, Breslow and Tarone-Ware tests for 5-year survival rate comparison according to the distribution of *E2F2* rs2075993 genotypes.


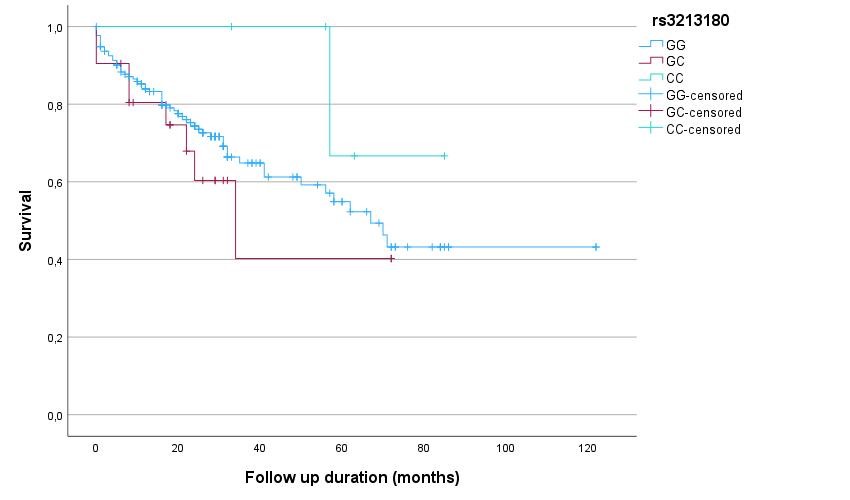


Figure S2. Five-year survival rate according to the distribution of *E2F1* rs3213180 genotypes.

|  | Chi-Square | df | p-value |
| --- | --- | --- | --- |
| Log Rank (Mantel-Cox) | 1.389 | 2 | 0.499 |
| Breslow (Generalized Wilcoxon) | 2.024 | 2 | 0.364 |
| Tarone-Ware | 1.943 | 2 | 0.379 |

Table S2. Log-rank, Breslow and Tarone-Ware tests for 5-year survival rate comparison according to the distribution of *E2F1* rs3213180 genotypes.


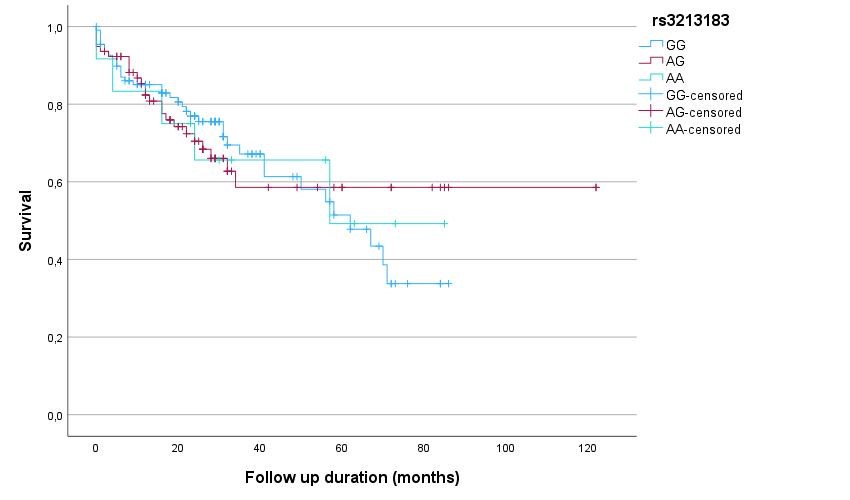


Figure S3. Five-year survival rate according to the distribution of *E2F1* rs3213183 genotypes.

|  | Chi-Square | df | p-value |
| --- | --- | --- | --- |
| Log Rank (Mantel-Cox) | 0.094 | 2 | 0.954 |
| Breslow (Generalized Wilcoxon) | 0.580 | 2 | 0.748 |
| Tarone-Ware | 0.340 | 2 | 0.844 |

Table S3. Log-rank, Breslow and Tarone-Ware tests for 5-year survival rate comparison according to the distribution of *E2F1* rs3213183 genotypes.


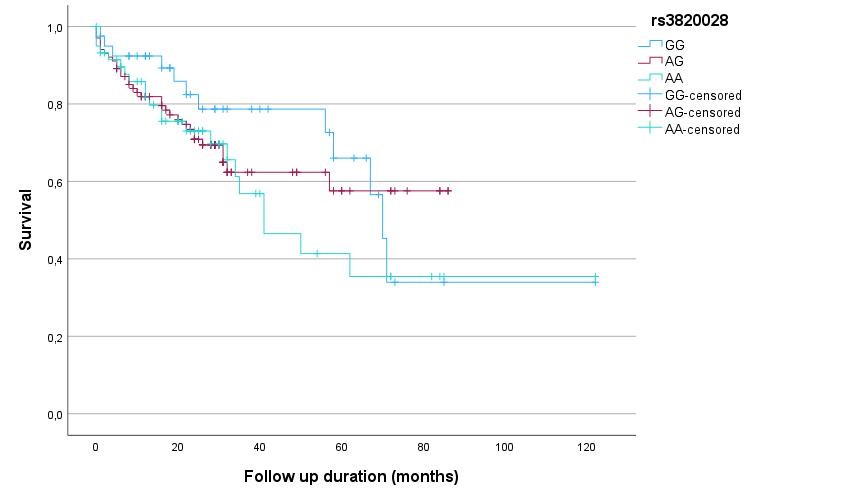


Figure S4. Five-year survival rate according to the distribution of *E2F2* rs3820028 genotypes.

|  | Chi-Square | df | p-value |
| --- | --- | --- | --- |
| Log Rank (Mantel-Cox) | 1.369 | 2 | 0.504 |
| Breslow (Generalized Wilcoxon) | 1.982 | 2 | 0.371 |
| Tarone-Ware | 1.841 | 2 | 0.398 |

Table 4. Log-rank, Breslow and Tarone-Ware tests for 5-year survival rate comparison according to the distribution of *E2F2* rs3820028 genotypes.
